# Effects of 6-month treatment with the glucagon like peptide-1 analogue liraglutide on arterial stiffness, left ventricular myocardial deformation and oxidative stress in subjects with newly diagnosed type 2 diabetes

**DOI:** 10.1186/s12933-017-0646-z

**Published:** 2018-01-08

**Authors:** Vaia Lambadiari, George Pavlidis, Foteini Kousathana, Maria Varoudi, Dimitrios Vlastos, Eirini Maratou, Dimitrios Georgiou, Ioanna Andreadou, John Parissis, Helen Triantafyllidi, John Lekakis, Efstathios Iliodromitis, George Dimitriadis, Ignatios Ikonomidis

**Affiliations:** 1 2nd Department of Internal Medicine, Research Unit and Diabetes Center, Attikon University Hospital, National and Kapodistrian University of Athens, Medical School, Rimini 1, Haidari, 12462 Athens, Greece; 2 2nd Cardiology Department, Attikon Hospital, National and Kapodistrian University of Athens, Medical School, Rimini 1 str, Haidari, 12462 Athens, Greece; 3 Hellenic National Center for the Prevention of Diabetes and Its Complications HNDC, 3 Ploutarchou str, 10675 Athens, Greece; 4 Department of Pharmaceutical Chemistry, School of Pharmacy, National and Kapodistrian University of Athens, Athens, Greece

**Keywords:** Arterial stiffness, Augmentation index, Glp-1 analogue, Liraglutide, Metformin, Left ventricular function, Oxidative stress

## Abstract

**Background:**

Incretin-based therapies are used in the treatment of type 2 diabetes mellitus (T2DM) and obesity. We investigated the changes in arterial stiffness and left ventricular (LV) myocardial deformation after 6-month treatment with the GLP-1 analogue liraglutide in subjects with newly diagnosed T2DM.

**Methods:**

We randomized 60 patients with newly diagnosed and treatment-naive T2DM to receive either liraglutide (n = 30) or metformin (n = 30) for 6 months. We measured at baseline and after 6-month treatment: (a) carotid-femoral pulse wave velocity (PWV) (b) LV longitudinal strain (GLS), and strain rate (GLSR), peak twisting (pTw), peak twisting velocity (pTwVel) and peak untwisting velocity (pUtwVel) using speckle tracking echocardiography. LV untwisting was calculated as the percentage difference between peak twisting and untwisting at MVO (%dpTw–Utw_MVO_), at peak (%dpTw–Utw_PEF_) and end of early LV diastolic filling (%dpTw–Utw_EDF_) (c) Flow mediated dilatation (FMD) of the brachial artery and percentage difference of FMD (FMD%) (d) malondialdehyde (MDA), protein carbonyls (PCs) and NT-proBNP.

**Results:**

After 6-months treatment, subjects that received liraglutide presented with a reduced PWV (11.8 ± 2.5 vs. 10.3 ± 3.3 m/s), MDA (0.92 [0.45–2.45] vs. 0.68 [0.43–2.08] nM/L) and NT-proBNP (p < 0.05) in parallel with an increase in GLS (− 15.4 ± 3 vs. − 16.6 ± 2.7), GLSR (0.77 ± 0.2 vs. 0.89 ± 0.2), pUtwVel (− 97 ± 49 vs. − 112 ± 52°, p < 0.05), %dpTw–Utw_MVO_ (31 ± 10 vs. 40 ± 14), %dpTw–Utw_PEF_ (43 ± 19 vs. 53 ± 22) and FMD% (8.9 ± 3 vs. 13.2 ± 6, p < 0.01). There were no statistically significant differences of the measured markers in subjects that received metformin except for an improvement in FMD. In all subjects, PCs levels at baseline were negatively related to the difference of GLS (r = − 0.53) post-treatment and the difference of MDA was associated with the difference of PWV (r = 0.52) (p < 0.05 for all associations) after 6-month treatment.

**Conclusions:**

Six-month treatment with liraglutide improves arterial stiffness, LV myocardial strain, LV twisting and untwisting and NT-proBNP by reducing oxidative stress in subjects with newly diagnosed T2DM.

ClinicalTrials.gov Identifier NCT03010683

## Background

Type-2 diabetes mellitus (T2DM) is associated with cardiac dysfunction [1]. Subclinical LV dysfunction is present in patients with T2DM and it is caused by factors such as insulin resistance, microvascular disease and cardiac autonomic dysfunction [2].

Glucagon-like peptide 1 (GLP-1) is an incretin hormone secreted mainly by the intestinal L-cells in response to the presence of nutrients [3]. GLP-1 analogues are a class of antidiabetic medications that mimic the actions of the endogenous incretin GLP-1 through supraphysiological blood concentrations that are resistant to degradation by DPP-4. These drugs lower glucose levels by inhibiting the secretion of glucagon, by promoting the release of insulin in a glucose-dependent manner, by slowing gastric emptying, and by acting at the hypothalamus causing an anorexigenic effect and thus regulating energy balance [4]. Recent large scale study has demonstrated that in addition to improving glycaemic control and promoting weight loss, GLP-1 analogues may improve cardiovascular outcomes in patients at high cardiovascular risk [5].

Liraglutide, an analogue of human GLP-1, has been approved for the treatment of T2DM and obesity [6, 7]. The long-term effects of liraglutide on cardiovascular outcomes were assessed in a recent study involving a high cardiovascular risk population including subjects with heart failure [5]. In the LEADER trial, cardiovascular benefit was observed after 1-year treatment with liraglutide, suggesting a beneficial effect in atherosclerosis, rather than an immediate effect on the haemodynamic parameters or the acute metabolic changes observed in the EMPA-REG trial [8].

Established prognostic markers of vascular integrity and function namely carotid-femoral pulse wave velocity (PWV), augmentation index (AI) and brachial artery flow-mediated dilation (FMD) have been found to be impaired in T2DM [9–11]. Myocardial deformation of the left ventricle (LV), as assessed by speckle tracking echocardiography, allows a sensitive assessment of LV function. Strain imaging is superior to LV ejection fraction in the detection of early type 2 diabetic myocardial disease [12, 13]. Abnormal PWV is related to impaired longitudinal LV function in hypertensive patients [14]. Measurement of natriuretic peptides like NT-proBNP contribute to the detection of left ventricular dysfunction [15]. Increased oxidative stress is a widely accepted participant in the development and progression of diabetes and its complications [16].

Study have shown that addition of GLP-1 analogue in patients with T2DM well controlled on metformin monotherapy improves several markers of vascular function [17]. However, the impact of treatment with GLP-1 analogue on vascular dysfunction, LV function, NT-proBNP and oxidative stress burden have not been clearly defined in patients with T2DM and no CVD history.

In the present study we hypothesized that a 6-month treatment with liraglutide in patients with newly diagnosed T2DM causes greater improvement in vascular function, LV myocardial strain, twisting–untwisting, oxidative stress burden as assessed by malondialdehyde (MDA) and protein carbonyls (PCs) and NT-proBNP concentrations compared to the standard treatment with metformin.

The aim of the present study was to investigate the early differences in vascular function, LV myocardial strain and twisting–untwisting and oxidative stress 6 months after treatment with either liraglutide or metformin in a population with a short disease duration and no prior therapeutic intervention.

## Methods

We examined 60 newly diagnosed and treatment-naive patients with type 2 diabetes mellitus. Patients were randomly assigned in a 1:1 ratio, to gradually receive either 1.8 mg of liraglutide once daily (with a weekly dose escalation as instructed by the SPC) as a subcutaneous injection or matching metformin 1000 mg twice daily for 6 months. Patients were recruited consecutively from the Diabetes Centre outpatient clinic. Exclusion criteria were history or clinical evidence of coronary or valvular heart disease, congestive heart failure, peripheral vascular disease, liver or kidney failure, history of alcohol or drug abuse, and treatments able to modify glucose metabolism. All women were premenopausal and their investigations were undertaken during the first week of their menstrual cycles. None of them were taking oral contraceptives. Dyslipidaemia was defined as total cholesterol > 220 mg/dl (LDL > 100 mg/dl and/or HDL < 40 mg/dl in men and HDL < 50 mg/dl in women) and/or triglycerides > 150 mg/dl. Hypertension was defined as clinic BP > 140/90 mmHg. Height, weight, body mass index (BMI) waist, and hips circumference were determined for all participants. 10 of the patients presented with various degrees of dyspnea at baseline and were classified as NYHA I stage of heart failure.

### Primary and secondary endpoints

The primary endpoint was a change in global longitudinal strain 6 months after treatment with either liraglutide or metformin.

Secondary endpoints were changes in endothelial function, arterial stiffness as assessed by pulse wave velocity and augmentation index. LV twisting and untwisting, NT-proBNP and oxidative stress post-treatment with either liraglutide or metformin.

### Blood pressure measurement

Each patient rested in a supine position for 10 min in a quiet room at 23 °C before the baseline haemodynamic measurements were recorded. Brachial blood pressure (BP) and heart rate (HR) were measured in the right arm with an automated digital oscillometric sphygmomanometer (TensioMed, Budapest Hungary, Ltd). Two sequential measurements separated by 2-min interval were obtained and the mean was used for statistical analysis.

### Assessment of arterial stiffness

At baseline and after 6-month treatment we measured the carotid-femoral pulse wave velocity (PWVc), central systolic blood pressure (cSBP), central pulse pressure (cPP) and augmentation index (AI). PWVc (m/s) was measured using tonometry by Complior (Alam Medical, Vincennes, France). Two non-invasive pressure sensors were used to record the carotid and femoral waveforms and the distance between the two arterial sites was measured with a tape measure. PWV was calculated as the distance divided by transit time between waves (m/s). Normal values for PWV < 10 m/s [18]. AI was defined as 100 × (P2 − P1)/PP where P2 = late backward systolic wave, P1 = early forward systolic wave, PP = pulse pressure, and represents the pressure boost that is induced by the return of the reflected waves at the aorta. AI_75_ was calculated to adjust the AI for a heart rate of 75 beats/min using the formula: AI_75_ = ([heart rate − 75] × 0.39) + AI [19]. The inter-and intra-observer variabilities for PWV were 6 and 5% and for Aix 12 and 10%, respectively.

### Endothelial function

Flow-mediated dilation (FMD) of the brachial artery was determined according to a previously published method and was expressed as a percentage change of the arterial diameter from the baseline vessel size [20]. Interobserver and intraobserver variability of the brachial artery diameter was, respectively, 0.08 ± 0.19 and 0.1 ± 0.12 mm, and the day-to-day variability of FMD was 1.1 ± 1%.

### Echocardiography

Studies were performed using a Vivid 7 (GE Medical Systems, Horten, Norway) ultrasound system. All studies were digitally stored in a computerized station (Echopac GE, Horten, Norway) and were analyzed by two observers blinded to clinical and laboratory data. For the determination of interobserver variability, data from the first 20 patients were analyzed by the 2 readers. Interobserver and intraobserver variabilities were calculated as the SD of the differences between the first and second measurements and expressed as a percentage of the average value.

### Doppler echocardiography

Using pulse-wave Doppler, E and A waves of the mitral inflow velocity were measured and the ratio E/A was calculated [20].

## 2D strain and strain rate analysis

In all patients, we measured longitudinal systolic strain (LS) and systolic strain rate (LSR) from standard 2-dimensional acquisitions (frame rate: 60–80/s) with the use of a dedicated software (EchoPac PC 201, GE Healthcare) [20]. Global longitudinal strain (GLS) and global longitudinal strain rate (GLSR) was calculated using the 17 LV segment model imaged from apical chamber views (4, 2 and 3 chamber view), as previously published [20, 21]. The intra- and inter-observer variability for LV strain parameters were 8 and 9% respectively. Normal values for GLS: − 20% [22].

### LV twisting and untwisting

Left ventricular rotation and twisting–untwisting were assessed using parasternal short axis views at basal and apical level [20, 21]. Subsequently, torsional deformation and velocity curves along with time were generated (EchoPac PC 201, GE Healthcare). Utilizing the pulse wave Doppler recording of mitral valve inflow, we measured time interval between the onset of the QRS of the simultaneous ECG recording and the onset, peak and end of the E wave of the mitral inflow waveform, respectively [20, 21]. Based on the above measured time intervals, utilizing as a starting point the onset of the QRS of the ECG recording in the generated torsional deformation curve along with time, we estimated peak twisting (pTw, deg), as well as untwisting at the time of mitral valve opening (Utw_MVO_), peak (Utw_PEF_) and end of left ventricular early filling (Utw_EDF_), respectively. The degree of LV untwisting during diastole was calculated as the percentage difference between peak twisting and untwisting at MVO (%dpTw–Utw_MVO_), at the peak (%dpTw–Utw_PEF_) and end of early filling (%dpTw–Utw_EDF_), using our previously published methodology [20, 21]. Furthermore, we measured peak twisting (pTw, deg), peak twisting velocity (pTwVel, deg/s) and peak untwisting velocity (pUtwVel, deg/s). The inter- and intra-observer variability these measurements were 8 and ≤ 10% respectively for all markers.

### Laboratory assays

Plasma glucose was measured by the enzymatic in vitro test (Roche, automatic chemistry clinical analyzer). Serum insulin concentration was determined by a chemiluminescense-based assay (Roche Diagnostics).

Malondialdehyde (MDA) and protein carbonyls (PCs) were determined spectrophotometrically with a commercial kit (Oxford Biomedical Research, Rochester Hills, MI) of colorimetric assay for lipid peroxidation (measurements range, 1–20 nmol/l) [23]. For the qualification of protein carbonyl content, we based on spectrophotometric measurement of 2,4-dinitrophenylhydrazine derivatives of protein carbonyls, as previously published [24] and results expressed as nmol/mg protein. Using commercially available enzyme-linked immunosorbent assay kits, we measured serum concentrations of amino-terminal Pro-B-Type Natriuretic Peptide (NT-proBNP) (ABNOVA, Taipei, Taiwan; sensitivity 2 pg/ml).

### Statistical analysis

All comparisons were performed with the Statistical Package for Social Sciences 21.0 for Windows (SPSS Inc., Chicago, Illinois, USA). Categorical data were compared between patients by contingency tables. Continuous variables were tested for normality using the Kolmogorov–Smirnov test. Normally distributed variables are given as mean ± standard deviation. Data with a non-gaussian distribution are expressed as median (interquartile range) and were analyzed after transformation into ranks. Differences in mean values for each of the measured variables were compared by t-test or paired t-test for continuous variables. For non-normally distributed data Mann–Whitney or Wilcoxon signed-rank test was used. We used parametric (Pearson r) and non-parametric (Spearman rho) correlation coefficients to examine cross-sectional associations. ANOVA (general linear model, SPSS 22, SPSS Inc, Chicago, Ill) for repeated measurements was applied (a) for measurements of the examined markers at baseline, 6 months after treatment used as a within-subject factor (b) for the effects of treatment (liraglutide vs. metformin), as a between-subject factors. The F and P values of the interaction between time of measurement of the examined markers and type of treatment were calculated. The F and P values of the comparison between treatments were calculated. The Greenhouse–Geisser correction was used when the sphericity assumption, as assessed by Mauchly’s test, was not met. Post hoc comparisons were performed with Bonferroni correction.

Comparisons between baseline or post-treatment values of measured markers between the two treatment groups were performed using factorial ANOVA. Post hoc comparisons were performed with Bonferroni correction.

Logistic regression analysis was performed to examine the association of the median values of GLS, PWV and FMD post-treatment with the type of study medication (liraglutide or metformin). Statistical significance was considered as p < 0.05. Baseline variables that were statistically different (p < 0.05) among the 2 study groups or were of clinical significance (HbA1c, weight, BMI and waist circumference) were included in multivariate models as covariates.

We planned to study the percent change (Δ) of GLS after treatment from independent control (patients on metformin) and experimental subjects (patients on liraglutide) with 1 control per experimental subject. In a pilot study of 10 patients treated with metformin and 10 treated with liraglutide, the response within each group was normally distributed with standard deviation 10%. The true difference between patients treated with metformin and those treated with liraglutide in the means of ΔGLS was 7.3%. Therefore, we would need to study 30 patients treated with metformin and 30 treated with liraglutide, to be able to reject the null hypothesis that the population means for ΔGLS post-treatment of the metformin and liraglutide groups are equal with probability (power) 0.8 and type I error probability 0.05.

## Results

The baseline characteristics for the study groups are shown in Table 1. Prior to the enrolment in the study, patients were not receiving diabetes medication and were being treated with diet and lifestyle therapy solely.

**Table 1:** Baseline characteristics of the study groups

	Total (n = 60)	Liraglutide (n = 30)	Metformin (n = 30)	p
Age, years	51 ± 12	51 ± 10	50 ± 12	0.594
Male sex, % (n)	66.7 (40)	66.7 (20)	66.7 (20)	0.896
Smoking, % (n)	36.6 (22)	36.7 (11)	36.7 (11)	0.782
Hypertension, % (n)	55 (33)	56.7 (17)	53.3 (16)	0.270
Dyslipidemia, % (n)	51.6 (31)	53.3 (16)	50 (15)	0.410
Family history CAD, % (n)	20 (12)	20 (6)	20 (6)	0.804
Creatinine, mg/dl	1.0 ± 0.2	1.1 ± 0.2	1.0 ± 0.3	0.733
eGFR, ml/min	85 ± 9	85 ± 8	83 ± 11	0.315
Medication				
Beta blockers, % (n)	18.3 (11)	16.7 (5)	20 (6)	0.492
Calcium antagonists, % (n)	30 (18)	33.3 (10)	26.7 (8)	0.231
ACE-I, ARB, % (n)	31.7 (19)	30 (9)	33.3 (10)	0.913
Diuretics, % (n)	13.3 (8)	13.3 (4)	13.3 (4)	0.984
Statins, % (n)	43.3 (26)	46.7 (14)	40 (12)	0.292

Data are expressed as the mean (SD) or n (%). p: p of the model of the ANOVA for comparisons between groups

### Changes in metabolic parameters and vascular markers after 6-month treatment

Treatment with liraglutide resulted in a greater weight loss, reduction in BMI, waist circumference, and HbA1c in comparison to metformin (p < 0.05, Table 2). Furthermore, treatment with liraglutide caused a significant reduction in PWV, AI, systolic blood pressure and central systolic blood pressure (p < 0.05, Tables 2 and 3) while these changes were not evident in patients treated with metformin (p > 0.05) after adjusting for HbA1c, weight, BMI and waist circumference. Thus, post treatment patients that received liraglutide had lower PWV, AI, systolic blood pressure and central systolic blood pressure than those on metformin (p < 0.05).

**Table 2:** Effect of treatment with liraglutide vs. metformin on anthropometric, biochemical and vascular variables

Time, months	Liraglutide (n = 30)			Metformin (n = 30)		
	0	6	p	0	6	p
Weight, Kg	98 ± 16	92 ± 16*	0.031	78 ± 12	77 ± 14	0.655
BMI, Kg/m^2^	32.9 ± 5	30.9 ± 5^#^	0.009	27.7 ± 2	26.9 ± 3	0.176
Waist, cm	109 ± 15	104 ± 14*	0.065	97 ± 12	95 ± 13	0.401
Fasting Glucose, mg/dl	165 ± 45	135 ± 33*	0.027	163 ± 40	143 ± 45	0.042
HbA1c, % (mmol/mol)	8.6 ± 2 (70 ± 22)	7 ± 1.2 (53 ± 13)*	0.006	8.4 ± 1.2 (68 ± 13)	7.7 ± 1 (61 ± 11)	0.012
SBP, mmHg	142 ± 15	138 ± 19*	0.024	142 ± 19	141 ± 16	0.632
DBP, mmHg	90 ± 8	87.5 ± 12	0.092	89 ± 9	88 ± 8	0.122
cSBP, mmHg	143 ± 20	138 ± 19*	0.035	142 ± 18	140 ± 18	0.574
HR, bpm	74 ± 12	80 ± 11*	0.043	71 ± 12	68 ± 10	0.075

Data are presented as mean ± SD values

*BMI* body mass index, *SBP* systolic blood pressure, *DBP* diastolic blood pressure, *cSBP* central systolic blood pressure, *HR* heart rate

* p < 0.05; ^#^p < 0.01—both for liraglutide vs. metformin post treatment

**Table 3:** Effect of treatment with liraglutide vs. metformin on arterial stiffness, LV function, and oxidative stress

Time, months	Liraglutide (n = 30)			Metformin (n = 30)		
	0	6	p	0	6	p
PWV, m/s	11.8 ± 2.5	10.3 ± 3.3*	0.019	11.2 ± 3	11 ± 3	0.719
AI_75_, %	18 (− 1 to 31)	13 (− 2 to 31)*	0.032	14 (− 9 to 24)	15 (− 8 to 24)	0.503
GLS, %	− 15.4 ± 3	− 16.6 ± 2.7*	0.043	− 15.5 ± 2.9	− 15.7 ± 3.2	0.721
GLSR, 1/s	0.77 ± 0.2	0.89 ± 0.2*	0.038	0.79 ± 0.3	0.82 ± 0.3	0.212
pTw, deg	15.5 ± 4	13.2 ± 6*	0.029	16.2 ± 5	15.0 ± 6	0.313
pUtw velocity, deg/s	− 97 ± 49	− 112 ± 52*	0.033	− 100 ± 41	− 98 ± 43	0.576
%dpTw–Utw_MVO_	31 ± 10	40 ± 14*	0.021	29 ± 18	30 ± 18	0.787
%dpTw–Utw_PEF_	43 ± 19	53 ± 22	0.018	45 ± 19	50 ± 16	0.874
E/A	0.92 ± 0.2	0.98 ± 0.3	0.555	0.99 ± 0.3	1.1 ± 0.4	0.679
FMD %	8.9 ± 3	13.2 ± 6*	0.003	8.8 ± 5	11.8 ± 6	0.033
MDA, nM/L	0.92 (0.45–2.45)	0.68 (0.43–2.08)^#^	0.006	0.78 (0.55–1.58)	0.86 (0.1–1.88)	0.09
PCs, nmol/mg protein	0.023 (0.011–0.026)	0.013 (0.008–0.017)	0.04	0.015 (0.006–0.019)	0.013 (0.009–0.017)	0.08
NT-proBNP, pg/ml	432 (154–2921)	282 (80–2302)*	0.03	490 (202–2670)	400 (98–2083)	0.08

Data are presented as mean ± SD values. Values for AI_75_ and biomarkers are median and interquartile range. PWV: pulse wave velocity; AI_75_ was calculated to adjust the AI for a heart rate of 75 beats/min using the formula: AI_75_ = ([heart rate − 75] × 0.39) + AI

*GLS* global longitudinal strain, *GLSR* global longitudinal strain rate, *pTw* peak twisting, *pUtw velocity* peak untwisting velocity, *%dpTw–Utw*_*MVO*_ percentage difference between peak twisting and untwisting at MVO, *%dpTw–Utw*_*PEF*_ percentage difference between peak twisting and untwisting at peak of left ventricular early filling, *E/A ratio* ratio of E to A waves of the mitral inflow velocity, *FMD%* percentage difference of flow mediated dilatation, *MDA* malondialdehyde, *PCs* protein carbonyls, *NT-proBNP* N-terminal pro-brain natriuretic peptide

ANOVA was adjusted for HbA1c, weight, BMI and waist circumference. * p < 0.05; ^#^p < 0.01—both for liraglutide vs. metformin post treatment

Heart rate was increased after liraglutide treatment in contrast to subjects who received metformin (p < 0.05, Table 2).

### Effect of treatment on endothelial function

Compared to baseline, FMD was increased in all subjects after 6-months treatment. However there was a significant interaction between type of treatment and change of FMD (p = 0.02). Among the two medications, liraglutide induced the greatest increase of percentage difference of FMD (Table 3) after adjusting for HbA1c, weight, BMI and waist circumference.

### Markers of oxidative stress and NT-proBNP

Compared to baseline MDA, PCs and NT-proBNP were decreased in all subjects after 6-months treatment. However, there was a significant interaction between type of treatment and change of MDA, PCs and NT-proBNP (p = 0.01, p = 0.03 and p = 0.03 respectively). Thus, treatment with liraglutide caused the greatest decrease of MDA, PCs and NT-proBNP (p < 0.05), while these changes were of borderline significance after metformin (Table 3) after adjusting for HbA1c, weight, BMI and waist circumference. Thus, post treatment patients that received liraglutide had lower MDA and NT-proBNP than those on metformin (p < 0.05).

### Speckle tracking analysis

#### Longitudinal strain

**Fig. 1:**
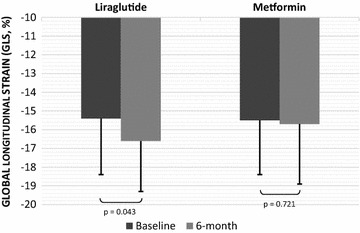
Changes in global longitudinal strain (GLS, %) after 6-month treatment with liraglutide or metformin. Data are presented as mean ± SD values

Compared to baseline, GLS and GLSR were increased after 6-month treatment with liraglutide (p < 0.05, Table 3, Fig. 1) after adjusting for HbA1c, weight, BMI and waist circumference. No significant changes were evident for GLS and GLSR in subjects that received metformin (p > 0.05, Table 3, Fig. 1). Thus, post treatment patients that received liraglutide had higher GLS than those on metformin (p = 0.03).

#### LV twisting and untwisting velocity

Compared to baseline, subjects under treatment with liraglutide showed a significant decrease in peak twisting and increase in peak untwisting velocity (p < 0.05) after adjusting for HbA1c, weight, BMI and waist circumference. In addition, the percentage changes between peak twisting and untwisting at mitral valve opening (%dpTw–Utw_MVO_) and peak (%dpTw–Utw_PEF_) of early diastolic LV filling were increased (p < 0.05, Table 3) suggesting a beneficial effect of liraglutide on LV twisting–untwisting. Metformin treatment did not cause any significant changes in the above parameter (p > 0.05, Table 3). Thus, post treatment patients that received liraglutide had lower peak twisting and peak untwisting velocity and higher  %dpTw–Utw_MVO_ than those on metformin (p < 0.05).

### Interrelation between metabolic, vascular, and LV function markers

In all subjects, at baseline GLS was positively related with HbA1c (b = 0.391, p = 0.01), PWV (b = 0.473, p < 0.001), SBP (b = 0.273, p = 0.032), and DBP (b = 0.351, p = 0.005) and negatively with FMD% (b = − 0.299, p = 0.043). In addition, GLSR was inversely related with HbA1c (b = − 0.352, p = 0.022), PWV (b = − 0.498, p < 0.001), SBP (b = − 0.308, p = 0.015), and DBP (b = − 0.407, p < 0.001). Furthermore, cSBP was associated with %dpTw–Utw_MVO_ (b = 0.293, p = 0.027).

After 6-month treatment, reduced PWV was positively related with GLS (b = 0.428, p = 0.026) and inversely with GLSR (b = − 0.488, p = 0.01) and FMD% (b = − 0.438, p = 0.047) in all subjects. In subjects that received liraglutide the percentage difference of HbA1c was associated with %dpTw–Utw_PEF_ (b = 0.642, p = 0.033).

In all subjects PCs levels at baseline was negatively related with the difference of GLS after 6 months (b = − 0.527, p = 0.023). Furthermore, after 6-months treatment the difference of MDA was associated with the difference of PWV (b = 0.522, p = 0.032). In addition, the difference of NT-proBNP was negatively associated with the difference of GLS post-treatment (b = − 0.39, p = 0.04).

Table 4 reports univariate and multivariable association of a PWV > 10 m/s, GLS > − 15% and FMD < 11% post treatment with type of medication (liraglutide vs. metformin) HbA1c, weight, BMI, waist, and heart rate. Compared to metformin, liraglutide treatment was related with a lower odds ratio for elevated PWV, reduced GLS and FMD (p < 0.05) in model including the above covariates.

**Table 4:** Univariate and multivariable association of PWV, GLS and FMD % post treatment with parameters of the study population

	PWV > 10 m/s				GLS < -15%				FMD > 11%			
	Univariate		Multivariable		Univariate		Multivariable		Univariate		Multivariable	
	Odds ratio	p	Odds ratio	p	Odds ratio	p	Odds ratio	p	Odds ratio	p	Odds ratio	p
HbA1c	1.499 (1.1–1.7)	0.035	1.709 (1.1–1.9)	0.047	1.104 (0.9–2)	0.085	1.668 (1–2.1)	0.033	1.456 (1.2–1.5)	0.030	1.422 (1.1–1.4)	0.048
Weight	1.037 (1–1.1)	0.096	1.200 (0.9–1.3)	0.059	1.099 (1–1.1)	0.067	1.172 (0.9–1)	0.029	1.225 (1–1.3)	0.039	1.054 (0.9–1.3)	0.057
BMI	1.142 (0.9–1.4)	0.071	1.241 (1.1–2)	0.092	1.325 (1–1.5)	0.065	1.490 (0.9–1.6)	0.014	1.198 (0.9–1.3)	0.042	1.304 (0.9–1.5)	0.072
Waist	1.252 (1–1.3)	0.045	1.077 (0.9–1.4)	0.062	1.132 (1–1.5)	0.037	1.516 (1–2)	0.043	1.427 (0.9–1.6)	0.033	1.873 (1–1.9)	0.011
HR	1.100 (1–1.2)	0.010	1.210 (1–1.4)	0.022	1.070 (1–1.3)	0.053	1.094 (1–1.3)	0.205	1.505 (1.1–1.6)	0.069	1.783 (1.2–1.9)	0.061
Liraglutide	0.333 (0.2–1.3)	0.018	0.367 (0.2–1.7)	0.046	0.815 (0.3–1.5)	0.043	0.920 (0.4–1.4)	0.046	0.650 (0.4–1.5)	0.035	0.790 (0.6–1.7)	0.049

The median values PWV, GLS and FMD of the makers were used in logistic regression analysis. *PWV* pulse wave velocity, *GLS* global longitudinal strain, FMD% percentage difference of flow mediated dilatation of brachial artery. Values are odd ratios (95% confidence intervals)

### Effect of weight loss on vascular and LV function markers

After 6-months treatment, liraglutide induced a greater decrease of weight than metformin (6% vs. 1.3%, p < 0.05). Subjects that received liraglutide and had a weight reduction of 1.3% (n = 10), which is identical to that caused by metformin presented reduced PWV (p = 0.021), AI_75_ (p = 0.04) and cSBP (p = 0.029) in parallel with an increased in GLS (p = 0.048), %dTw–Utw_MVO_ (p = 0.031) and FMD% (p = 0.023) compared to baseline. These results show that the better effects of liraglutide treatment compared to metformin on arterial stiffness, LV myocardial deformation and endothelial function are not simply an effect of weight loss.

## Discussion

In the present study, treatment with liraglutide for 6 months led to a significant reduction of arterial stiffness, oxidative stress burden and NT-proBNP level in parallel with an improvement of LV longitudinal myocardial strain and strain rate, LV twisting–untwisting and endothelial function as assessed by FMD in newly diagnosed, treatment-naive T2DM patients. These changes were not evident after treatment with metformin. Thus patients that received liraglutide had better LV longitudinal deformation twisting and untwisting than those on metformin. Additionally, the reduction of oxidative stress was associated with improved arterial elasticity as assessed by PWV which in turn was associated with improved myocardial deformation after 6 months of treatment. Finally and the increase of LV longitudinal deformation post treatment was to a reduction of NT-proBNP levels.

The spatial organization of myocardial fibers has a major impact in cardiac mechanics. Longitudinal LV deformation is mainly attributed to subendocardial fibers [25]. In terms of LV twisting, the subepicardial fibers, by their larger radius, produce the dominant force for LV rotation, whereas the subendocardial torque partly counteracts this twist. Consequently, impaired function of subendocardial myocardial helix results in: (i) the predominance of the function of the subepicardial helix during systole leading to increased LV twisting, and (ii) the impairment of myocardial relaxation in diastole leading to delayed untwisting in diastole [20, 25]. Thus, improved longitudinal deformation by a direct effect of liraglutide on cardiomyocytes has led to the reduction of an abnormally augmented LV twisting as observed in our study.

### Incretin-based therapies and direct cardiovascular effects

So far, the direct effects of incretin-based therapies on myocardial contractility and endothelial function have been controversial. Various theories have been put forward to explain the protective effects of GLP-1 on myocardium. The most upheld revolves around cardiac metabolism [26]. GLP-1 analogues, have been shown to ameliorate insulin resistance and inflammation [27, 28]. They also seem to promote an actual increase in glucose transporters GLUT-2 and GLUT-4, especially in cardiomyocytes [29, 30].

A 3-months’ exenatide delivery to sedentary patients with uncomplicated, although not treatment-naive, type 2 diabetes, has recently shown to improve cardiac function and reduce arterial stiffness, although it proved neutral in terms of functional exercise capacity and endothelial function [31]. Conversely, postprandial haemodynamics were assessed after acute and chronic treatment with GLP1 analogs (exenatide infusion vs. placebo and liraglutide vs. sitagliptin or placebo, respectively) in a double-blind, placebo-controlled randomized trial. The study showed that a 12-week liraglutide administration was neutral in terms of postprandial decrease in diastolic blood pressure, which is thought to impair coronary perfusion in type 2 diabetic patients, as well as in other indices of postprandial haemodynamics. However, the study was a substudy and not all patients had postprandial hypotension at baseline, nor did they all have measurements at week 12 due to protocol design. The treatment dose was not generally stable, and administration route was different among GLP1 agonists, which might also have interfered with the results [32].

In another randomized, double-blind, placebo-controlled crossover study, liraglutide failed to show benefit in the systolic function or the exercise capacity of patients with preserved ejection fraction, and stable coronary artery disease. However, the study population had already undergone revascularization leading to a low probability of abnormal stress response anyway. Interestingly, the largest improvement in ejection fraction in the present study was observed during recovery period after peak stress which agrees with our results [33].

In addition, a lack in FMD change with liraglutide was recently exhibited in a study of 16 DM patients already on antidiabetic treatment (e.g. DDP-4) during a 14 week follow up period compared to the respective FMD change of 15 patients treated with insulin [34]. The greater change of FMD post GLP-1 treatment in our study could be attributed: (a) to the longer treatment period of our study (6 months vs. 14 and 20 weeks, respectively) (b) to the fact that our patients were naïve to antidiabetic treatment and thus there was no carry on effect of previous medications on endothelial function while in the study by Nomoto more than 40% were receiving DDP4 inhibitor and all patients were treated with antidiabetic medication prior to inclusion in the study (c) the FMD examination in our study was performed within the tmax of liraglutide injection (9–12 h) while in the study by Nomoto et al. this was performed a day after the last injection of GLP-1 [34].

Sitagliptin has also failed to confirm a beneficial net effect on pulse wave velocity within 2 years of therapy. However, the results suggested that achievement of good glycaemic control inhibits the annual progression of the arterial stiffness [35].

GLP-1 analogues have additional direct natriuretic effect and action on endothelial vasodilatation [36, 37]. A meta-analysis assessing the effect of GLP-1 based therapy on FMD showed neutral results in longitudinal studies within a 20 weeks’ period though there was a trend of increased FMD values post-treatment [38]. Conversely, within the same study, when a meta-analysis of 7 cross-sectional studies was conducted, results showed a large increase in FMD values post GLP-1 based treatment [38].

Even asymptomatic diabetic patients with preserved LV ejection fraction present with complications that are closely associated with systolic dysfunction [39]. Arterial stiffness is a determinant of LV longitudinal strain and twisting–untwisting by affecting perfusion of subendocardial myocardial fibres [21]. Ιn our study, a decreased afterload post-liraglutide treatment due to the reduced brachial systolic and central systolic blood pressure and decreased arterial stiffness may have contributed to the increased myocardial perfusion, reduced oxygen demand and consequently to improved LV function [20, 21, 23]. Indeed, lower values of PWV post treatment were related to the respective improvement of GLS and GLSR.

Previous reporting of elevated heart rate associated with liraglutide treatment was also observed in our study [7]. The chronotropic effect of GLP-1 analogues are believed to be mediated via GLP-1 receptors located to the sinoatrial node [40]. However, GLP-1 may exhibit an inhibitory effect on sympathovagal balance as well [41]. This increase in heart rate has not outweighed the cardioprotective effects of liraglutide as assessed with the significant improvement in myocardial deformation and reduction of NT-proBNP after liraglutide treatment in our study.

### Effects of weight reduction and glycaemic control

There were no effects of metformin on vascular and LV myocardial deformation, in spite of a similar glycaemic control as assessed by a comparable HbA1c post-treatment between patients treated with liraglutide and metformin. Thus the observed changes in biochemical and vascular markers should not be attributed to differences in glycaemic status post treatment.

In our study liraglutide caused a greater decrease of weight than metformin. However, a subgroup of patients that received liraglutide and had a weight reduction identical to that caused by metformin treatment had also improved arterial stiffness and myocardial deformation post-treatment. These results show that the better effects of liraglutide treatment compared to metformin on arterial stiffness, LV myocardial deformation and endothelial function should not be attributed solely to weight loss. Indeed, in multivariable analysis including BMI, weight, waist circumference and HbA1c, liraglutide treatment predicted an improved GLS (> − 15%), FMD (> 11%) and PWV (< 10 m/s) post treatment.

### Effects on oxidative stress markers

Malondialdehyde and PCs are valid biomarkers of lipid peroxidation and protein oxidation respectively [42]. Glycation has been reported to induce the formation of protein carbonyls, such as ketoamine derivatives, thus generating reactive radicals and perpetuating a vicious cycle [43]. Cytokines and nitro-oxidative stress have a direct negative inotropic effect and promote myocardial ischemia, apoptosis, and LV dysfunction [20, 44]. Therefore, increased oxidative stress leading to vascular dysfunction and fibrosis may explain the link between arterial stiffness and impairment of LV myocardial deformation [45]. The interplay between oxidative stress, inflammation, endothelial dysfunction and type 2 diabetes have recently been studied in a multicenter, longitudinal investigation of the evolution of cardiovascular disease risk starting in young adulthood. Biomarkers of inflammation and endothelial dysfunction were positively associated with incident type 2 diabetes. Specifically, ICAM-1 and E-selectin added to the prediction of T2D beyond a common risk score [46].

In line with the above, in a 26-week, randomized, open-label, two-arm, parallel-group study, compared treatment with insulin glargine vs. exenatide on top of other standard of care therapies. Although both agents improved glycaemia and endothelial function, incretin therapy was superior for weight management, and several cardiovascular biomarkers’ profile, mainly cytokines like hs-CRP, MCP-1, fibrinogen and endothelin-1, implying a more pronounced anti-inflammatory effect [47]. The same was true for teneligliptin, a novel DPP4 inhibitor, that was found to ameliorate endothelial function and to reduce renal and vascular oxidative stress in patients with type 2 diabetes and chronic kidney disease, irrespectively of reducing albuminuria or glycaemia [48].

In our study baseline PCs levels determined the percent reduction of PWV post treatment. Furthermore, the reduction of oxidative stress, as assessed by MDA, was associated with improved arterial elasticity as assessed by PWV which in turn was associated with improved myocardial deformation after 6 months of treatment. The decrease of MDA and PCs was greater after liraglutide than after metformin treatment. Thus a lower oxidative stress level resulted in a greater improvement of vascular function and LV myocardial deformation.

### Effects on natriuretic peptides

Natriuretic peptides are produced primarily within the heart and released into the circulation in response to increased wall tension [49]. Circulating concentrations of N-terminal pro-brain natriuretic peptide (NT-proBNP) are raised in both symptomatic and asymptomatic patients with left ventricular dysfunction [50]. In our study, reduced NT-proBNP post treatment was associated with increased LV longitudinal strain. Again, the reduction of NT-proBNP was greater after liraglutide than after metformin treatment in parallel with the greater beneficial effect of liraglutide on LV longitudinal deformation.

### Study limitations and strengths

Οur study has a modest number of subjects. Additional large scale studies are needed to expand our results. In addition, our study could be extended in time in order to investigate the long-term benefits from the use of GLP-1 analogues on the cardiovascular system and endothelial function and determine how long can these benefits be maintained.

The present study adds to the literature, as it involves diabetic patients early in the course of disease, without prior treatment to interfere with the results, and also without clinical cardiovascular disease. It shows a beneficial effect of liraglutide in specific cardiac biomarkers and non-invasive measurements of cardiac function as well as in oxidative stress within solely 6-months. The latter suggests that this treatment category might benefit younger patients with a classic cardiometabolic profile earlier, before overt cardiovascular disease develops. Of note, in the LEADER trial it took 1 year for the treatment to show an overall CVD benefit, but without a specific beneficial effect on myocardial infarction (silent infarctions were not included in the analysis) [5]. Thus, one could conclude that if type 2 diabetic patients with the classic atherogenic profile are treated earlier in the course of disease with such agents, myocardial and endothelial function could be preserved. As the current study is a mechanistic study, whose aim is to show the beneficial effects of liraglutide on strict surrogate markers of cardiovascular function it does not suggest that liraglutide is a first-line treatment in DM.

## Conclusions

In the present study, 6-month treatment with liraglutide resulted in a greater improvement of endothelial function, arterial stiffness, LV myocardial strain, twisting and untwisting, NT-proBNP and oxidative stress than metformin in newly diagnosed and treatment-naive patients with T2DM. Thus, in diabetic patients with evidence of arterial stiffness and impaired LV deformation even subclinically, early targeted antidiabetic treatment which offers myocardial protection may retard the progression to diabetic heart disease.

## Abbreviations

T2DM: type 2 diabetes mellitus
LV: left ventricle
GLP-1: glucagon like peptide-1
PWV: pulse wave velocity
GLS: global longitudinal strain
GLSR: global longitudinal strain rate
pTw: peak twisting
pTwVel: peak twisting velocity
pUtwVel: peak untwisting velocity
%dpTw–Utw_MVO_: the percentage difference between peak twisting and untwisting at mitral valve opening
%dpTw–Utw_PEF_: the percentage difference between peak twisting and untwisting at peak of early LV diastolic filling
%dpTw–Utw_EDF_: the percentage difference between peak twisting and untwisting at end of early diastolic filling
FMD: flow mediated dilatation
FMD%: the percentage difference of flow mediated dilatation
MDA: malondialdehyde
PCs: protein carbonyls
NT-proBNP: N-terminal pro-brain natriuretic peptide
